# Supplementary figures and images for: Discrimination between Onchocerca volvulus and O. ochengi filarial larvae in Simulium damnosum (s.l.) and their distribution throughout central Ghana using a versatile high-resolution speciation assay
Source: Parasit Vectors. 2016 Oct 10;9:536. doi: 10.1186/s13071-016-1832-7 (PMC5057476; doi:10.1186/s13071-016-1832-7)

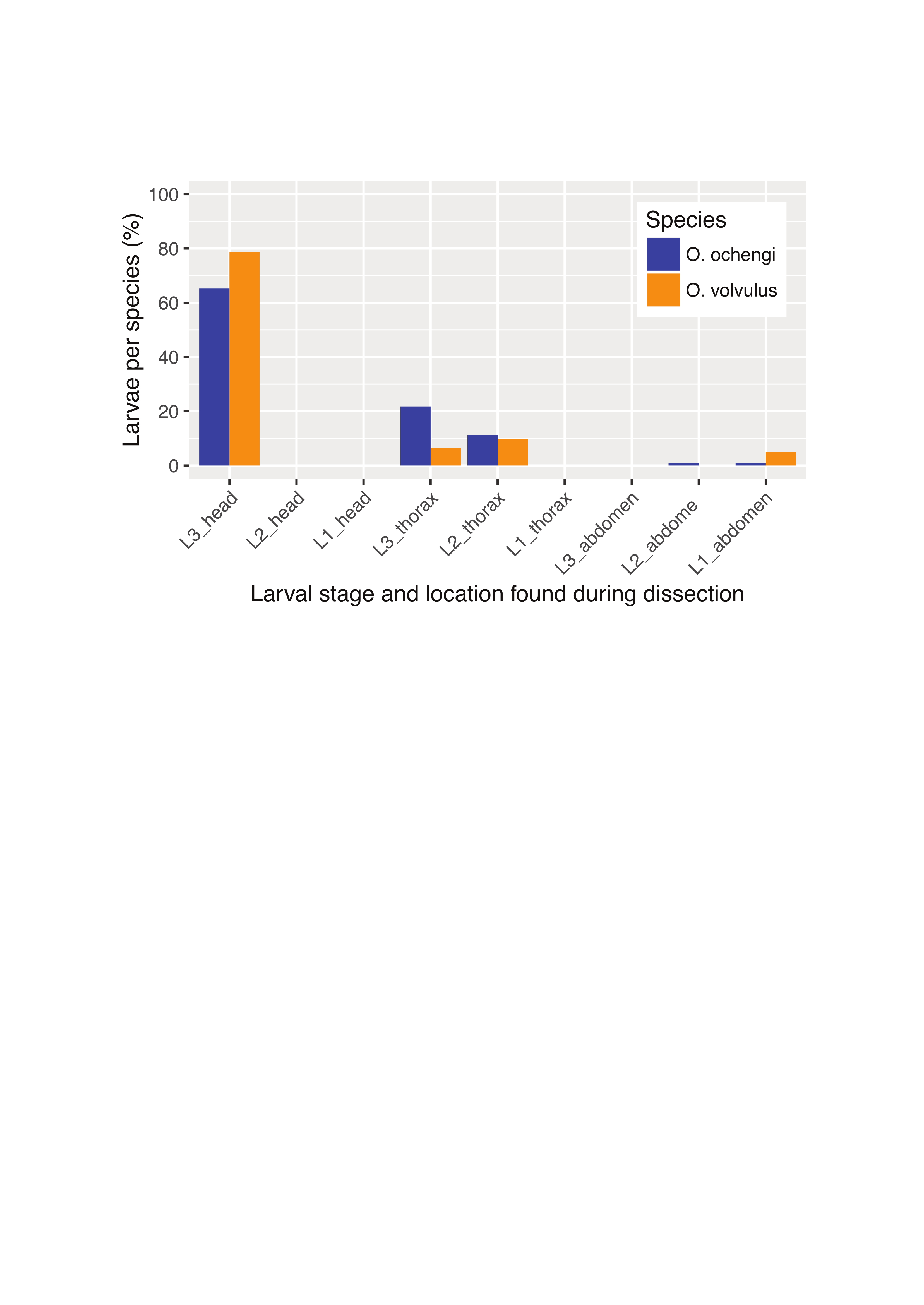

Supplement: Additional file 2: Figure S1. — Proportion of O. volvulus (orange) and O. ochengi (purple) larval life stages (L3, L2, & L1) found during dissection of the blackfly head, thorax and abdomen. Values reported are a percentage of the total larvae found within each species. (TIF 60491 kb) [file 13071_2016_1832_MOESM2_ESM.tif]
